# Radiographic Position of Impacted Mandibular Third Molars and Their Association with Pathological Conditions

**DOI:** 10.1155/2021/8841297

**Published:** 2021-03-24

**Authors:** Zahra Haddad, Mansour Khorasani, Mahin Bakhshi, Maryam Tofangchiha, Zeynab shalli

**Affiliations:** ^1^Dental Faculty, Qazvin University of Medical Sciences, Qazvin, Iran; ^2^Department of Oral & Maxillofacial Surgery. Dental Faculty, Tehran University of Medical Sciences, Tehran, Iran; ^3^Department of Oral Medicine, Dental Faculty, Shahid Beheshti University of Medical Sciences, Tehran, Iran; ^4^Department of Oral and Maxillofacial Radiology, Dental Caries Prevention Research Center, Qazvin University of Medical Sciences, Qazvin, Iran; ^5^Department of Orthodontics, Dental Faculty, Qazvin University of Medical Sciences, Qazvin, Iran

## Abstract

**Objectives:**

This study aimed to assess the radiographic position of impacted mandibular third molars (IMTMs) and their association with pathological conditions.

**Materials and Methods:**

The impaction depth, relationship with ramus, and angulation of 1600 IMTMs and their association with 2^nd^ molar distal caries and root resorption, pathological conditions, and proximity to the mandibular canal were evaluated on panoramic radiographs. The IMTM position was determined based on the depth of impaction according to the Pell and Gregory classification, relationship with ramus according to the Pell and Gregory classification, and angulation according to the Winter's classification. The classical and Bayesian logistic regressions were applied to analyze the effect of IMTM position on the associated complications using the odds ratio (OR) and 95% confidence interval (credible interval for Bayesian models). Two-tailed *P* value < 0.05 was considered statistically significant.

**Results:**

Of 1600 IMTMs evaluated in this study, 195 (12.2%), 252 (15.8%), and 119 (7.4%) had caused second molar distal caries, second molar root resorption, and pathological lesions, respectively, and 872 (54.5%) had contact with the mandibular canal. Impaction angulation was a risk factor for second molar distal caries (maximum OR = 5.01, 95% CI: 3.12–8.18). Changed angulation and greater impaction depth were the risk factors for second molar root resorption (minimum OR = 1.64, 95% CI: 0.58–4.02). Decreased distance between the ramus and distal side of the second molar was a risk factor for associated pathological lesions (minimum OR = 2.73, 95% CI: 1.79–4.25). Mesioangular and horizontal angulations and greater impaction depth were the risk factors for contact with the mandibular canal (maximum OR = 3.44, 95% CI: 2.6–4.57 and minimum OR = 1.3, 95% CI: 094–1.8).

**Conclusions:**

The frequency of complications associated with IMTMs was low, but considerable. The occurrence of these conditions might be affected by the impaction position. Thus, regular follow-ups are recommended in order to be able to surgically intervene when the first signs of pathologies arise.

## 1. Introduction

Mandibular third molars are the most commonly impacted teeth, which are usually impacted due to different reasons. The two major causes of impaction include (I) failure of tooth rotation from the horizontal to mesioangular and vertical position and (II) space shortage for eruption [1]. Impacted mandibular third molars (IMTMs) can cause various pathological complications such as pericoronitis, periodontal disease, distal caries, bone loss, root resorption of the adjacent teeth, odontogenic cysts and tumors, jaw fractures, and infections [1–13].

Third molar surgery is among the most common surgical procedures worldwide. This surgical procedure may be indicated for the following purposes: (I) to treat the symptomatic pathologies related to impaction, (II) to prevent future pathological conditions, or (III) for non-pathological reasons, e.g., prior to orthognathic surgery, orthodontic treatment, and removable denture treatment [14]. Despite the necessity of removal of impacted third molars in the abovementioned situations, their preventive removal is still a matter of controversy.

Hundreds of patients undergo third molar surgery every year. However, the real benefit of this procedure is still a matter of debate, and the advantages and disadvantages of prophylactic third molar tooth removal should be weighed against the treatment of subsequent complications in case of their development. In this respect, various strategies have been adopted by different countries. Preventive removal has been rejected in the UK by the National Institute of Clinical Excellence [15], while there is controversy in the United States on this topic between the American Association of Oral and Maxillofacial Surgeons (AAOMS) and the American Public Health Association (APHA). AAOMS believes in encouraging prophylactic removal of third molars in young ages to prevent future problems and to ensure optimal healing. On the other hand, APHA disagrees with the prophylactic removal of third molars, because of its unnecessary costs for patients and the society, avoidable morbidity, and the risks of permanent injury [16, 17]. Moreover, in Germany and Japan, third molars are removed if their physiological eruption is not expected [18, 19]. Thus, a general consensus is lacking on this topic.

Although the National Institute of Clinical Excellence, AAOMS, and APHA have published guidelines for management of impacted third molars, AAOMS calls for further studies to determine the prevalence of pathological conditions associated with impacted third molars [20].

It appears that information about the pathological conditions associated with third molar impaction plays a key role in compiling a comprehensive guideline regarding third molar management. Hence, the aim of this study was to radiographically evaluate the impaction status of impacted mandibular third molars (impaction depth, relation with the ascending ramus, and angulation) and the associated pathological conditions.

## 2. Materials and Methods

### 2.1. Study Design/Sample

To address the research question, a retrospective cross-sectional study was conducted on panoramic radiographs of patients referred to a private oral and maxillofacial radiology clinic in Qazvin city, Iran.

**Figure 1:**
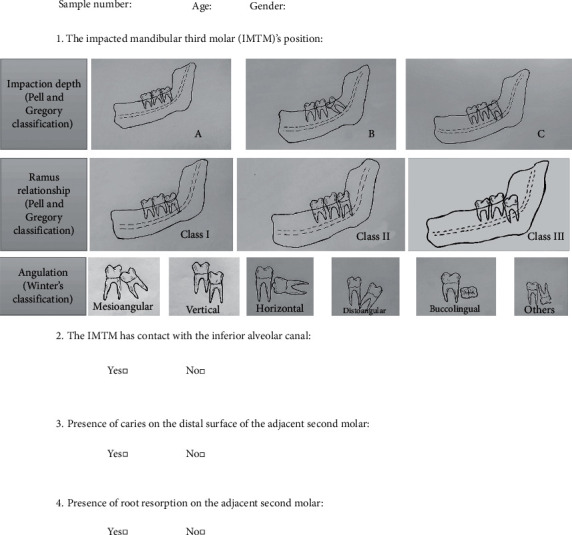
The questionnaire used in the study.

At the onset of the study, in order to calibrate the examiners for data collection and to enhance data recording, a checklist was designed under the supervision of an oral and maxillofacial surgeon with 20 years of clinical experience (Figure 1). The examiners included an oral and maxillofacial radiologist with 10 years of clinical experience, and a senior dental student.

High-quality digital panoramic radiographs of patients were retrieved from the archives. The images had been obtained by Cranex 3D x-ray system (Soredex, Finland) with the standard exposure parameters of 70 kVp, 16 mA, and 15 s. The magnification rate ranged from 0% to 50%.

The radiographs were selected by convenience sampling.

Patients were excluded if they met any of the following conditions: age under 20 years (since the eruption of third molars usually starts at this age) [21], previous history of trauma to the jaw involving the dentition, craniofacial anomalies (for example, Down syndrome), mandibular third molars with incomplete root formation, erupted mandibular third molars, mandibular third molars with less than half of their distal cusp in the ascending ramus, missing mandibular third molars, missing mandibular second molars, and missing mandibular first molars resulting in tilting of the adjacent mandibular second molar.

### 2.2. Study Variables

**Figure 2:**
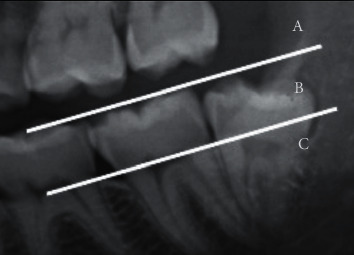
The impaction depth classified according to the Pell and Gregory classification with respect to the occlusal plane. Class A: the highest part of the mandibular third molar is located on the same level or above the occlusal plane of the adjacent second molar. Class B: the highest part of the mandibular third molar is located between the occlusal plane and the cervical line of the second molar. Class C: the highest part of the mandibular third molar is located below the cervical line of the second molar.

**Figure 3:**
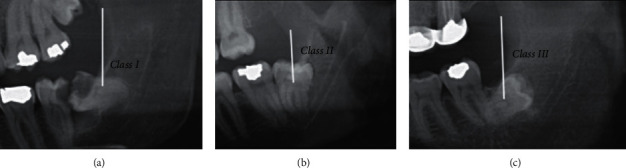
Pell and Gregory classification of ramus relation. (a) Class I: sufficient space available between the anterior border of the ascending ramus and distal side of the second molar for third molar eruption. (b) Class II: the space available between the anterior border of the ascending ramus and distal side of the second molar is less than the mesiodistal width of third molar's crown. It indicates that ascending ramus bone is covering the distal portion of the third molar crown. (c) Class III: absolute lack of space is observed; the third molar is totally embedded in the ascending ramus bone.

**Figure 4:**
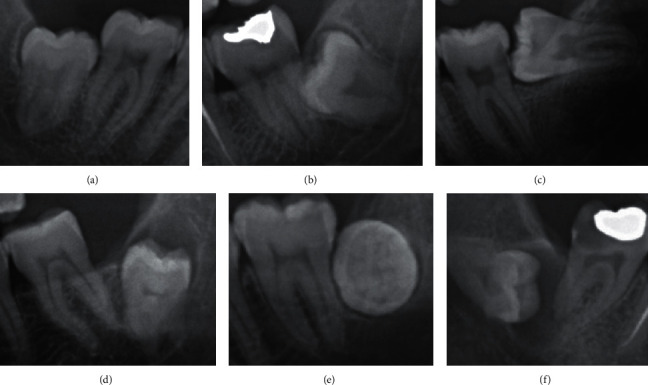
Angulation of impaction according to the Winter's classification. (a) Vertical impaction: 10° to −10°. (b) Mesioangular impaction: 11° to 79°. (c) Horizontal impaction: 80° to 100°. (d) Distoangular impaction: −11° to −79°. (e) Buccolingual impaction: when the crown and roots are superimposed. (f) Others: 111° to −80°.

Independent variables evaluated in this study were depth of impaction classified according to the Pell and Gregory classification (Figure 2), relation with the ramus classified according to the Pell and Gregory classification (Figure 3) [22], and angulation of IMTMs according to the Winter's classification (Figure 4) [23].

**Figure 5:**
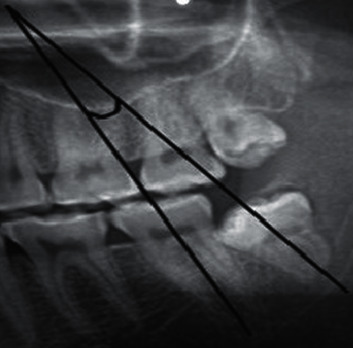
Method of angulation measurement. The angle formed at the intersection of the two lines was measured by an orthodontic protractor.

The longitudinal axis of mandibular molars was determined as the line connecting the midpoint of the occlusal surface and bifurcation of the tooth. The angle formed between the longitudinal axis of the second and third molars was measured by an orthodontic protractor (Figure 5).

Dependent variables evaluated in this study were presence of caries in the distal surface of the adjacent second molar, root resorption of the second molar, proximity to the mandibular canal, and presence of intra-bony pathological lesions related to the IMTM.

In order to distinguish distal caries from distal root resorption, discontinuity and irregularity of the root surface and loss of tooth structure at the cervical region, lateral wall, or apex was considered as root resorption [24], while a distal radiolucency with relation to the oral environment and a gap between the third and second molars that caused food impaction indicated the presence of distal caries [4].

**Figure 6:**
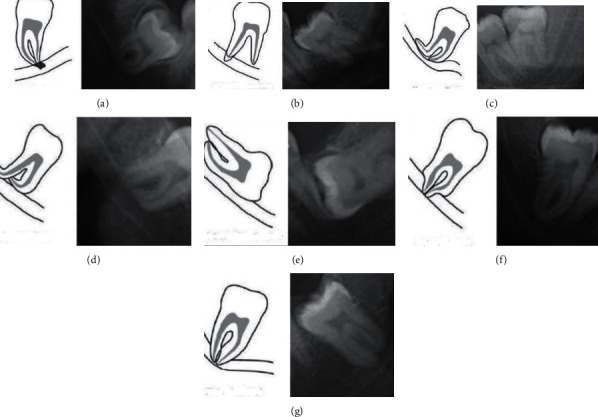
Radiographic signs showing close relation between the mandibular third molar and the mandibular canal. (a) Darkening of root. (b) Dark and bifid apex of root. (c) Narrowing of the canal. (d) Deflection of root. (e) Interruption of white line of the canal. (f) Narrowing of root. (g) Diversion of canal.

Proximity to the mandibular canal was assessed by using the Rood and Shehab radiographic markers that indicate close relationship between the IMTM and the mandibular canal (Figure 6) [25]. According to Meyer's classification for the correlation of third molars with the mandibular canal, “notching of the root” is a condition where the mandibular canal is in close physical contact with an indentation in the lateral side of the root [26]. Accordingly, IMTMs were categorized into three groups of “in contact with the inferior alveolar nerve (IAN),” “no contact with the IAN,” and “in contact with the external border of the mandibular canal.”

**Figure 7:**
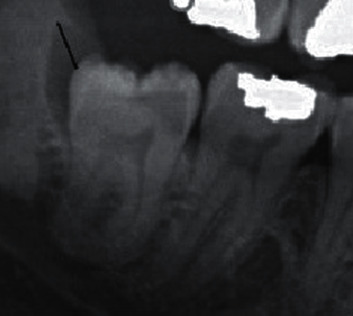
Any radiolucency around the impacted tooth that was larger than 3 mm was considered as a pathological lesion.

In the present study, any radiolucency, e.g., follicular hyperplasia, odontogenic cysts, or tumors related to the IMTMs, or any reduction in bone density around the IMTMs was considered as a pathological lesion [10]. Any radiolucency around the impacted tooth that was larger than 3 mm was considered as a pathological lesion (Figure 7).

### 2.3. Data Collection

Data were collected over a 9-month period. The images were stored on a hard disk drive and were evaluated in a dark room on a SONY VPCCW laptop computer. The checklist was once filled out by a senior dental student, and then all items were double-checked by a radiologist. If there was any disagreement, both examiners discussed the case with each other until a consensus was reached. Therefore, no inter-examiner error was expected. A third molar was considered impacted if it had no functional occlusion with completely formed roots [1]. The radiographs were evaluated anonymously. The study protocol was approved by the ethics committee of Qazvin University of Medical Sciences (QUMS.REC.1394.30).

### 2.4. Statistical Analysis

Classical and Bayesian logistic regressions were used to analyze the effects of IMTM position on pathological conditions by using the odds ratio (OR) and 95% confidence interval (credible interval for Bayesian models). Two-tailed *P* values < 0.05 were considered statistically significant. Statistical analyses were performed using *R* software (version 4.0.1).

## 3. Results

A total of 2018 panoramic radiographs of 2832 teeth were evaluated, out of which 1011 panoramic images were entered in the study. A total of 589 patients had bilateral IMTMs; therefore, 1600 IMTMs of patients between 20 and 55 years (mean age of 25.73 ± 5.26 years) were evaluated.

The frequency of mesioangular position (36%) was higher than other positions; moreover, class B (46.8%) and class II (51.3%) were the most frequent impaction positions.  Table 1 shows the frequency of different impaction positions and ramus relation of IMTMs according to the Pell and Gregory classification.

Of 1600 IMTMs evaluated in this study, 872 (54.5%) had contact with the internal space of the mandibular canal, 207 (12.9%) had contact with the mandibular canal borders, and 521 (32.6%) had no contact with the canal. Also, 252 (15.8%) IMTMs had caused second molar root resorption, 195 (12.2%) had caused distal caries in the adjacent second molar, and 119 (7.4%) cases had pathological lesions.

A strong correlation was found between the depth of impaction (according to the Pell and Gregory classification) and second molar distal root resorption (minimum OR = 4.78, 95% CI: 3.31–7.08, *P* < 0.001), second molar distal caries (maximum OR = 0.24, 95% CI: 0.17–0.34, *P* < 0.001), third molar pathological lesions (maximum OR = 0.54, 95% CI: 0.28–0.98, *P* ≤ 0.001), and contact with the mandibular canal (minimum OR = 2.2, 95% CI: 1.52–3.22, *P* ≤ 0.001, Table 2).

Class A position had the strongest association with second molar caries and pathological lesions. Moreover, class B and class C both had higher correlation with contact with the mandibular canal and distal root resorption (Table 2).

A strong relationship existed between the ramus relation (according to the Pell and Gregory classification) and presence of complications (Table 3). Second molar distal caries and root resorption were more frequent in class I position, while IMTM pathological lesions had the highest association with class Ш position (OR = 8.07, 95% CI: 2.76–20.84, *P* < 0.001) and class III had higher association with contact with the mandibular canal (OR = 1.34, 95% CI: 0.56–3.75, *P*=0.535) (Table 3).

Change in angulation of third molar from the vertical position had the highest correlation with second molar distal root resorption (minimum OR = 1.64, 95% CI: 0.58–4.02, *P* < 0.305). Second molar distal caries and contact with the mandibular canal had the highest correlation with mesioangular and vertical positions. The least correlation was with pathological lesions (Table 4).

Table 5 shows the odds of contact of IMTM root with the mandibular canal border, and interference of the IMTM root with the internal mandibular canal space based on different positions of IMTMs.

## 4. Discussion

This study provided new evidence for the management of IMTMs according to the correlation of impaction position in three dimensions (tooth angulation, Pell and Gregory occlusogingival position, and Pell and Gregory mesiodistal position) and some complications.

### 4.1. Relationship between Distal Caries and IMTM Position

In the present study, distal caries was observed in 195 (12.2%) second molars out of 1600 teeth investigated. As panoramic radiography may have lower diagnostic accuracy for detection of proximal caries [27], 12.2% rate of second molar distal caries can only be an estimation of the actual prevalence of distal caries, and the frequency rate reported in the present study is probably an underestimation of the actual rate. However, Rushton et al. [28] declared that panoramic radiography is appropriate for detection of clinically evident carious lesions. The incidence of second molar distal caries is reportedly 7%–42.7% in the literature [3, 8–10, 29]. Variations in the reported rates may be related to the age range of patients, cultural and socioeconomic differences of the study populations, their oral hygiene habits, the diagnostic methods used, and differences in assessing the second molar distal caries in one or both jaws. To the best of the authors' knowledge, the correlation of mandibular third molar impaction position and second molar distal caries and the effect of different positions and angulations have not been adequately studied.

Falci et al. [3] reported that second molar distal caries occurs more frequently in class II, followed by class I and class III IMTMs. In the present study, class I IMTMs exhibited the strongest association with second molar distal caries (62.6%), followed by class II (37.4%) and class III (0%) IMTMs. This finding indicates that second molar distal caries may be absent when an IMTM is fully embedded in the ramus bone, as there is no contact between the IMTM and the adjacent tooth. Significant differences between class I impaction versus class II and class III impactions reinforce the idea that plaque accumulation and food impaction increase the risk of second molar distal caries.

A strong correlation was found between the impaction depth (Pell and Gregory classification) and second molar distal caries in the present study, since 75.4% of IMTMs causing second molar distal caries had class A impaction, 24.1% had class B impaction, and 1% had class C impaction. By an increase in depth of impaction, risk of second molar caries decreased (OR = 0.24, 95% CI: 0.17–0.34, *P* < 0.001 for class B and OR = 0.04, 95% CI: 0.01–0.14, *P* < 0.001 for class C). This finding was in accordance with the findings of other studies [2, 3, 29]. Plaque accumulation and food impaction have higher frequency in IMTMs with class A position due to their close contact with the second molar. In contrast with the present study, Falci et al. [3] found no significant correlation between impaction depth and distal caries.

Chung et al. [29] found 27 caries-free class C third molars in their study population. They discussed that a class C third molar is completely impacted and its crown is below the cervical line of the second molar; thus, it cannot cause second molar distal caries. Contrary to the findings of Chung et al. [29], one class C IMTM had caused distal caries in the adjacent tooth in our study. This particular case was a horizontal IMTM having a contact point with the cementoenamel junction (CEJ) of the adjacent tooth causing an extensive carious lesion starting in the second molar's crown and extending down and involving the coronal third of the distal root. In such cases, the angulation of tooth and the location of contact point must be considered. Ozec et al. [30] reported that a contact point on the CEJ of the second molar had a statistically significant effect on development of distal caries. We also found that tooth angulation had a significant effect on development of distal caries in the adjacent tooth. In our study, 74.4% of all distal caries occurred in presence of mesioangular and horizontal IMTMs, in a decreasing order of frequency (OR = 3.2, 95% CI: 2.11–4.99, *P* < 0.001, and OR = 5.01, 95% CI: 3.12–8.18, *P* < 0.001, respectively). This finding was in agreement with other studies [3, 30].

### 4.2. Second Molar Root Resorption and IMTM Position

Second molar root resorption caused by the pressure applied by the third molar has been explained in different studies [4, 8, 20, 24, 31]. The incidence of second molar root resorption is 0.3%–9.5% [4, 6, 8–10, 31, 32]. The highest incidence of second molar root resorption was reported by Nemcovsky et al. [33]. In the present study, the frequency of second molar root resorption was 15.8%, which is considerable. One probable explanation for the variations in the reported frequency rates of root resorption could be the different definitions of root resorption. In some studies, root resorption was defined as a clear loss of root structure of the adjacent second molar [4, 6, 8, 20, 34], while the present study and some others considered discontinuity and irregularity of the root surface and loss of tooth structure as root resorption [24, 31].

To the best of the authors' knowledge, the correlation of mandibular third molar impaction position and second molar root resorption and the effect of different positions and angulations have not been adequately studied. Only three studies were found to report the most frequent impaction position and angulation of third molars [4, 33, 34], and two evaluated IMTM position [20, 24]. The current results confirmed that occlusal surface position of IMTMs according to the Pell and Gregory classification, tooth position in relation to ramus according to the Pell and Gregory classification, and tooth angulation were associated with development of this pathological condition. This could explain the different results of studies since one previous study reported root resorption caused by completely impacted mandibular third molars [24], while another study reported that partially impacted mandibular third molars were more capable of causing adjacent root resorption [20]. In this study, changing the angulation and greater depth of impaction relative to the reference level increased the risk of second molar root resorption, while changing the ramus relation with IMTM relative to the reference level decreased root resorption. A horizontal IMTM, class I ramus relation, and class C impaction level had the greatest association with distal root resorption of the adjacent second molar.

### 4.3. Relationship between Pathological Lesions and IMTM Position

As bone density reduction was the most common pathological lesion investigated in this study, it was found that vertical, class Ш, and class C IMTMs had caused more pathological lesions. The most important factor responsible for development of pathological lesions was the IMTM position relative to the ramus such that any change from the reference level increased the risk of development of pathological lesions. However, with regard to depth of impaction and angulation, any change from the reference level decreased the risk of pathological lesions. Polat et al. reported that the prevalence of bone loss at the distal aspect of IMTM was 9.7% and it was found in association with distoangular and vertical IMTM positions [2].

### 4.4. Relationship between IAN Contact and IMTM Position

The position and root location of IMTMs are among the most important factors associated with IAN damage [26, 35]. In the present study, seven radiographic markers were used concurrently [19, 25, 35–40]. These markers were related to impaction depth and ramus relation according to the Pell and Gregory classification, and angulation of IMTMs [19]. The results of the present study revealed that 54.5% of IMTMs had contact with the mandibular canal, while 12.9% had contact with the external border of the canal. As contact with the mandibular canal can cause IAN neurosensory deficits after third molar surgery, a frequency of 54.5%–67.4% of contact with the canal can be considerable. Thus, in case of presence of signs of contact of IMTM with the mandibular canal, another imaging method such as cone-beam computed tomography should be requested [41–43], although the sensitivity and specificity of cone-beam computed tomography were reported to be 93% and 73%, respectively, for this purpose [44].

Although class I and class II IMTMs showed higher frequency in terms of relation with the mandibular canal, class III had higher odds to be associated with the mandibular canal such that contact with the mandibular canal was observed in 75% of class III IMTMs (18 of 24 class III IMTMs), due to the anatomical position of the mandibular canal. Although class A IMTMs had higher frequency of contact with the mandibular canal, as expected, class B and C IMTMs had higher odds of contact with the mandibular canal. Mesioangular IMTMs showed greater contact with the mandibular canal, which was in agreement with other studies [19, 45, 46]. By an increase in impaction depth and changing the IMTM angulation from vertical to mesioangular and horizontal, risk of interference of the IMTM root with the mandibular canal increased. Also, class III cases had higher odds of interference with the mandibular canal than class I by 34%. By an increase in impaction depth, the odds of interference with the mandibular canal (contact with the canal wall and contact with the internal space) increased. IMTMs in horizontal position and class III IMTMs had significantly higher risk of interference of their root with the IAN, compared with contact with the external border of the canal; in other words, IMTMs in horizontal position and class III IMTMs had significantly higher risk of interference with the IAN and lower risk of contact with the external canal wall. In total, the frequency of complications associated with IMTMs was low but considerable in our study.

The occurrence of related complications with IMTMs is affected by the depth and angulation of impacted teeth. Therefore, identifying IMTMs with higher risk of related complications can lead to early surgical intervention as soon as the first signs of pathologies arise; hence, regular follow-ups are strongly recommended for such cases.

Long-term studies are suggested to evaluate the exact clinical and radiographic characteristics that favor retaining of asymptomatic IMTMs against their prophylactic extraction. Regular clinical and radiographic follow-ups are necessary until a comprehensive guideline is reached on this topic.

## 5. Conclusion

With regard to IMTM angulation, the frequency of all complications increased in mesioangular and horizontal positions except for pathological lesions. Thus, it may be concluded that mesioangular and horizontal positions are associated with higher risk of such complications. Distoangular position was also associated with higher risk of all complications, except for interference with the mandibular canal. In assessment of ramus relation, classes II and III were associated with higher risk of pathological lesions. Also, class III had higher risk of interference with the mandibular canal. In assessment of impaction depth, greater impaction depth was associated with higher risk of interference with the mandibular canal and root resorption.

## Figures and Tables

**Table 1:** Frequency distribution and percentage of independent variables.

	Frequency *n* (%)
Pell and Gregory classification (occlusal surface)	
A	684 (42.8)
B	749 (46.8)
C	167 (10.4)
Total	1600 (100)
Pell and Gregory classification (ramus)	
I	756 (47.3)
II	820 (51.3)
III	24 (1.5)
Total	1600 (100)
Winter classification (angulation)	
Vertical	535 (33.4)
Mesioangular	576 (36)
Horizontal	231 (14.4)
Distoangular	111 (6.9)
Buccolingual	28 (1.8)
Others	119 (7.4)
Total	1600 (100)

**Table 2:** Frequency and percentage of complications based on impaction depth of mandibular third molars.

*Pell and Gregory (occlusal surface)*	Root resorption			Distal caries			Pathologic lesions			Relation with the IAC		
	*n* (%)	OR (95% CI)	*P* value	*n* (%)	OR (95% CI)	*P* value	*n* (%)	OR (95% CI)	*P* value	*n* (%)	OR (95% CI)	*P* value
A^*∗*^	36 (2.2)	1	—	147 (9.2)	1	—	85 (5.3)	1	—	379 (23.7)	1	—
B	157 (9.8)	4.78 (3.31,7.08)	<0.001	47 (2.2)	0.24 (0.17,0.34)	<0.001	22 (1.4)	0.21 (0.13,0.34)	<0.001	578 (36.1)	2.72 (2.17,3.41)	<0.001
C	59 (3.7)	9.74 (6.17,15.58)	<0.001	1 (0.1)	0.04 (0.01,0.14)	<0.001	12 (0.8)	0.54 (0.28,0.98)	0.057	122 (7.6)	2.2 (1.52,3.22)	<0.001

^*∗*^A is considered as the reference level.

**Table 3:** Frequency and percentage of complications based on relationship with the mandibular ramus.

Pell and Gregory (ramus)	Root resorption			Distal caries			Pathologic lesions			Relation with the IAC		
	*n* (%)	OR (95% CI)	*P* value	*n* (%)	OR (95% CI)	*P* value	*n* (%)	OR (95% CI)	*P* value	*n* (%)	OR (95% CI)	*P* value
I^*∗*^	167 (10.4)	1	—	122 (7.6)	1	—	30 (1.9)	1	—	522 (32.6)	1	—
II	82 (5.1)	0.39 (0.29,0.52)	<0.001	73 (4.6)	0.51 (0.38,0.7)	<0.001	83 (5.2)	2.73 (1.79,4.25)	<0.001	539 (33.7)	0.86 (0.7,1.06)	0.161
III	3 (0.2)	0.5 (0.12,1.48)	0.272	0 (0)	0.08 (0,1.3)	0.077	6 (0.4)	8.07 (2.76,20.84)	0.057	18 (1.2)	1.34 (0.56,3.75)	0.535

^*∗*^I is considered as the reference level.

**Table 4:** Frequency and percentage of complications based on the angulation of impacted mandibular third molars.

Winter classification on angulation	Root resorption			Distal caries			Pathologic lesions			Relation with the IAC		
	*n* (%)	OR (95% CI)	*P* value	*n* (%)	OR (95% CI)	*P* value	*n* (%)	OR (95% CI)	*P* value	*n* (%)	OR (95% CI)	*P* value
Vertical^*∗*^	18 (1.1)	1	—	30 (1.9)	1	—	65 (4.1)	1	—	332 (20.1)	1	—
Mesioangular	131 (8.2)	8.46 (5.22,14.52)	<0.001	92 (5.8)	3.2 (2.11,4.99)	<0.001	23 (1.4)	0.3 (0.18,0.48)	<0.001	483 (30.2)	3.44 (2.6,4.57)	0.001
Horizontal	55 (3.4)	8.98 (5.23,16.1)	<0.001	53 (3.3)	5.01 (3.12,8.18)	<0.001	5 (0.3)	0.16 (0.06,0.37)	<0.001	153 (9.6)	1.3 (0.94,1.8)	0.114
Distoangular	6 (0.4)	1.64 (0.58,4.02)	0.305	10 (0.6)	1.67 (0.75,3.41)	0.18	18 (1.1)	1.4 (0.77,2.42)	0.246	62 (3.9)	0.84 (0.55,1.27)	0.398
Buccolingual	4 (0.2)	4.79 (1.31,14.06)	0.008	1 (0.1)	0.62 (0.03,3.09)	0.648	2 (0.1)	0.56 (0.09,1.92)	0.431	6 (0.4)	0.18 (0.07,0.43)	<0.001
Others	38 (2.4)	13.47 (7.44,25.25)	<0.001	9 (0.6)	1.38 (0.6,2.87)	0.417	6 (0.4)	0.38 (0.15,0.84)	0.029	106 (6.6)	0.53 (0.35,0.79)	0.002

^*∗*^Vertical is considered as the reference level.

**Table 5:** Frequency and percentage of the relation of mandibular third molars with different positions with the mandibular canal.

Position of IMTMs (depth and angulation)	Within IAC (contact with IAN)			Contact with IAC's external border		
	*n* (%)	OR (95% CI)	*P* value	*n* (%)	OR (95% CI)	*P* value
Vertical^*∗*^	240 (15)	1	—	82 (5.1)	1	—
Mesioangular	409 (25.6)	3.9 (2.93,5.24)	<0.001	74 (4.6)	2.07 (1.39,3.08)	<0.001
Horizontal	134 (8.4)	1.52 (1.09,2.14)	0.013	19 (1.2)	0.63 (0.35,1.09)	0.111
Distoangular	46 (2.9)	0.83 (0.53,1.3)	0.419	16 (1)	0.85 (0.45,1.55)	0.602
Buccolingual	3 (0.2)	0.12 (0.03,0.36)	0.001	3 (0.2)	0.35 (0.08,1.06)	0.099
Others	40 (2.5)	0.54 (0.35,0.83)	0.005	66 (4.1)	0.51 (0.26,0.95)	0.042
A^*∗*^	290 (18.1)	1	—	89 (5.6)	1	—
B	484 (30.2)	2.97 (2.35,3.77)	<0.001	94 (5.9)	1.88 (1.33,2.66)	<0.001
C	98 (6.1)	2.31 (1.58,3.43)	<0.001	24 (1.5)	1.83 (1.04,3.14)	0.031
I^*∗*^	427 (26.7)	1	—	95 (5.9)	1	—
II	427 (26.7)	0.83 (0.67,1.04)	0.102	112 (7)	0.99 (0.72,1.36)	0.934
III	18 (1.1)	1.64 (0.68,4.59)	0.299	0 (0)	0.14 (0.01,2.54)	0.18

^*∗*^Vertical, A, and I are considered as the reference level.

## Data Availability

The data used to support the findings of this study are available from the corresponding author upon request.

## References

[1] Hupp J. R., Ellis E., Tucker M. R. (2014). *Contemporary Oral and Maxillofacial Surgery*.

[2] Polat H. B., Ozan F., Kara I., Ozdemir H., Ay S. (2008). Prevalence of commonly found pathoses associated with mandibular impacted third molars based on panoramic radiographs in Turkish population. *Oral Surgery, Oral Medicine, Oral Pathology, Oral Radiology, and Endodontology*.

[3] Falci S. G. M., de Castro C. R., Santos R. C. (2012). Association between the presence of a partially erupted mandibular third molar and the existence of caries in the distal of the second molars. *International Journal of Oral and Maxillofacial Surgery*.

[4] Oenning A. C. C., Neves F. S., Alencar P. N. B., Prado R. F., Groppo F. C., Haiter-Neto F. (2014). External root resorption of the second molar associated with third molar impaction: comparison of panoramic radiography and cone beam computed tomography. *Journal of Oral and Maxillofacial Surgery*.

[5] McArdle L. W., McDonald F., Jones J. (2014). Distal cervical caries in the mandibular second molar: an indication for the prophylactic removal of third molar teeth? Update. *British Journal of Oral and Maxillofacial Surgery*.

[6] Akarslan Z. Z., Kocabay C. (2009). Assessment of the associated symptoms, pathologies, positions and angulations of bilateral occurring mandibular third molars: is there any similarity?. *Oral Surgery, Oral Medicine, Oral Pathology, Oral Radiology, and Endodontology*.

[7] Patil S., Halgatti V., Khandelwal S., Santosh B. S., Maheshwari S. (2014). Prevalence of cysts and tumors around the retained and unerupted third molars in the Indian population. *Journal of Oral Biology and Craniofacial Research*.

[8] Al-Khateeb T. H., Bataineh A. B. (2006). Pathology associated with impacted mandibular third molars in a group of Jordanians. *Journal of Oral and Maxillofacial Surgery*.

[9] Chu F. C., Li T. K., Lui V. K., Newsome P. R., Chow R. L., Cheung L. K. (2003). Prevalence of impacted teeth and associated pathologies—a radiographic study of the Hong Kong Chinese population. *Hong Kong Medical Journal = Xianggang yi xue za zhi / Hong Kong Academy of Medicine*.

[10] Linden W. V. D., Cleaton-Jones P., Lownie M. (1995). Diseases and lesions associated with third molars. *Oral Surgery, Oral Medicine, Oral Pathology, Oral Radiology, and Endodontology*.

[11] Bezerra T. P., Studart-Soares E. C., Pita-Neto I. C., Costa F. W., Batista S. H. (2011). Do third molars weaken the mandibular angle?. *Medicina Oral Patología Oral y Cirugia Bucal*.

[12] Krimmel M., Reinert S. (2000). Mandibular fracture after third molar removal. *Journal of Oral and Maxillofacial Surgery*.

[13] Meisami T., Sojat A., Sàndor G. K. B., Lawrence H. P., Clokie C. M. L. (2002). Impacted third molars and risk of angle fracture. *International Journal of Oral and Maxillofacial Surgery*.

[14] Steed M. B. (2014). The indications for third-molar extractions. *The Journal of the American Dental Association*.

[15] NICE Guidance (2000). https://www.nice.org.uk/guidance/ta1.

[16] APHA (2008). *Policy Statements and Advocacy*.

[17] PogrelMA S. J., Bonine F. L. (2014). https://www.aaoms.org/practice-resources/aaoms-advocacy-and-position-statements/white-papers/.

[18] Hasegawa T., Ri S., Shigeta T. (2013). Risk factors associated with inferior alveolar nerve injury after extraction of the mandibular third molar-a comparative study of preoperative images by panoramic radiography and computed tomography. *International Journal of Oral and Maxillofacial Surgery*.

[19] Szalma J., Lempel E., Jeges S., Szabó G., Olasz L. (2010). The prognostic value of panoramic radiography of inferior alveolar nerve damage after mandibular third molar removal: retrospective study of 400 cases. *Oral Surgery, Oral Medicine, Oral Pathology, Oral Radiology, and Endodontology*.

[20] Oenning A. C. C., Sousa Melo S. L., Groppo F. C., Haiter-Neto F. (2015). Mesial inclination of impacted third molars and its propensity to stimulate external root resorption in second molars-A cone-beam computed tomographic evaluation. *Journal of Oral and Maxillofacial Surgery*.

[21] Blaeser B. F., August M. A., Donoff R. B., Kaban L. B., Dodson T. B. (2003). Panoramic radiographic risk factors for inferior alveolar nerve injury after third molar extraction. *Journal of Oral and Maxillofacial Surgery*.

[22] Pell G. J., Gregory G. T. (1942). Report on a ten-year study of a tooth division technique for the removal of impacted teeth. *American Journal of Orthodontics and Oral Surgery*.

[23] Winter G. (1926). The principles of exodontias as applied to the impacted third molar. *American Medical book (ed) Management of Impacted Teeth*.

[24] Yamaoka M., Furusawa K., Ikeda M., Hasegawa T. (1999). Root resorption of mandibular second molar teeth associated with the presence of the third molars. *Australian Dental Journal*.

[25] Rood J. P., Nooraldeen Shehab B. A. A. (1990). The radiological prediction of inferior alveolar nerve injury during third molar surgery. *British Journal of Oral and Maxillofacial Surgery*.

[26] Meyer R. A., Bagheri S. C. (2011). Nerve injuries from mandibular third molar removal. *Atlas of the Oral and Maxillofacial Surgery Clinics*.

[27] Kamburoglu K., Kolsuz E., Murat S., Yüksel S., Ozen T. (2012). Proximal caries detection accuracy using intraoral bitewing radiography, extraoral bitewing radiography and panoramic radiography. *Dento Maxillo Facial Radiology*.

[28] Rushton V. E., Horner K., Worthington H. V. (2002). Routine panoramic radiography of new adult patients in general dental practice: relevance of diagnostic yield to treatment and identification of radiographic selection criteria. *Oral Surgery, Oral Medicine, Oral Pathology, Oral Radiology, and Endodontology*.

[29] Chang S. W., Shin S. Y., Kum K. Y., Hong J. (2009). Correlation study between distal caries in the mandibular second molar and the eruption status of the mandibular third molar in the Korean population. *Oral Surgery, Oral Medicine, Oral Pathology, Oral Radiology, and Endodontology*.

[30] Özeç İ., Hergüner Siso Ş., Taşdemir U., Ezirganli Ş., Göktolga G. (2009). Prevalence and factors affecting the formation of second molar distal caries in a Turkish population. *International Journal of Oral and Maxillofacial Surgery*.

[31] Nitzan D., Keren T., Marmary Y. (1981). Does an impacted tooth cause root resorption of the adjacent one?. *Oral Surgery, Oral Medicine, Oral Pathology*.

[32] Kahl B., Gerlach K. L., Hilgers R.-D. (1994). A long-term, follow-up, radiographic evaluation of asymptomatic impacted third molars in orthodontically treated patients. *International Journal of Oral and Maxillofacial Surgery*.

[33] Nemcovsky C. E., Libfeld H., Zubery Y. (1996). Effect of non-erupted 3rd molars on distal roots and supporting structures of approximal teeth A radiographic survey of 202 cases. *Journal of Clinical Periodontology*.

[34] Knutsson K., Brehmer B., Lysell L., Rohlin M. (1996). Pathoses associated with mandibular third molars subjected to removal. *Oral Surgery, Oral Medicine, Oral Pathology, Oral Radiology, and Endodontology*.

[35] Sanmartí-Garcia G., Valmaseda-Castellón E., Gay-Escoda C. (2012). Does computed tomography prevent inferior alveolar nerve injuries caused by lower third molar removal?. *Journal of Oral and Maxillofacial Surgery*.

[36] Shahidi S., Zamiri B., Bronoosh P. (2013). Comparison of panoramic radiography with cone beam CT in predicting the relationship of the mandibular third molar roots to the alveolar canal. *Imaging Science in Dentistry*.

[37] Guerrero M. E., Botetano R., Beltran J., Horner K., Jacobs R. (2014). Can preoperative imaging help to predict postoperative outcome after wisdom tooth removal? A randomized controlled trial using panoramic radiography versus cone-beam CT. *Clinical Oral Investigations*.

[38] Sedaghatfar M., August M. A., Dodson T. B. (2005). Panoramic radiographic findings as predictors of inferior alveolar nerve exposure following third molar extraction. *Journal of Oral and Maxillofacial Surgery*.

[39] Leung Y. Y., Cheung L. K. (2011). Correlation of radiographic signs, inferior dental nerve exposure, and deficit in third molar surgery. *Journal of Oral and Maxillofacial Surgery*.

[40] Leung Y. Y., Cheung L. K. (2011). Risk factors of neurosensory deficits in lower third molar surgery: a literature review of prospective studies. *International Journal of Oral and Maxillofacial Surgery*.

[41] Ebrahimifard T., Poorzamani M., Tavakoli M., Varshowsaz M. (2013). The validity of the panoramic radiography in evaluating the topographic relationship between mandibular canal and impacted third molars in comparison with cone beam CT-scan. *Zahedan Journal of Research in Medical Sciences*.

[42] Palma-Carrio C., Garcia-Mira B., Larrazabal-Moron C., Penarrocha-Diago M. (2010). Radiographic signs associated with inferior alveolar nerve damage following lower third molar extraction. *Medicina Oral Patología Oral y Cirugia Bucal*.

[43] Neves F. S., Souza T. C., Almeida S. M., Haiter-Neto F., Freitas D. Q., Bóscolo F. N. (2012). Correlation of panoramic radiography and cone beam CT findings in the assessment of the relationship between impacted mandibular third molars and the mandibular canal. *Dentomaxillofacial Radiology*.

[44] Tantanapornkul W., Okouchi K., Fujiwara Y. (2007). A comparative study of cone-beam computed tomography and conventional panoramic radiography in assessing the topographic relationship between the mandibular canal and impacted third molars. *Oral Surgery, Oral Medicine, Oral Pathology, Oral Radiology, and Endodontology*.

[45] Selvi F., Dodson T. B., Nattestad A., Robertson K., Tolstunov L. (2013). Factors that are associated with injury to the inferior alveolar nerve in high-risk patients after removal of third molars. *British Journal of Oral and Maxillofacial Surgery*.

[46] Blondeau F., Daniel N. G. (2007). Extraction of impacted mandibular third molars: postoperative complications and their risk factors. *Journal (Canadian Dental Association)*.

